# Green Synthesis and Urease Inhibitory Activity of Spiro-Pyrimidinethiones/Spiro-Pyrimidinones-Barbituric Acid Derivatives

**Published:** 2015

**Authors:** Ghodsi Mohammadi Ziarani, Shima Asadi, Sakineh Faramarzi, Massoud Amanlou

**Affiliations:** a *Research Laboratory of Pharmaceutical, Alzahra University, Tehran, Iran. *; b *Drug Design and Development Research Center and Department of Medicinal Chemistry, Faculty of Pharmacy, Tehran University of Medical Sciences, Tehran, Iran.*

**Keywords:** Biginelli like reaction, Spiro heterobicyclic rings, SBA-Pr-SO_3_H, Multicomponent reaction (MCR), Urease inhibitory, Barbituric acid

## Abstract

Sulfonic acid functionalized SBA-15 (SBA-Pr-SO_3_H) with pore size 6 nm as an efficient heterogeneous nanoporous solid acid catalyst exhibited good catalytic activity in the Biginelli-like reaction in the synthesis of spiroheterobicyclic rings with good yield and good recyclability. Spiro-pyrimidinethiones/spiro-pyrimidinones-barbituric acid derivatives were synthesized in a simple and efficient method using the one-pot three-component reaction of a cyclic 1,3- dicarbonyl compounds (barbituric acid), an aromatic aldehyde and urea or thiourea in the presence of nanoporous silica SBA-Pr-SO_3_H under solvent free conditions. Urease inhibitory activity of spiro compounds were tested against Jack bean urease using Berthelot alkaline phenol–hypochlorite method. Five of 13 compounds were inhibitor and two of them were enzyme activators. Analysis of the docking results showed that, in most of the spiro molecules, one of the carbonyl groups is coordinated with both nickel atoms, while the other one is involved in the formation of hydrogen bonds with important active-site residues. The effect of inserting two methyl groups on N atoms of barbiturate ring, S substituted, *ortho, meta* and *para* substituted compounds were investigated too.

## Introduction

Many hundreds of chemical compounds across the world are tested daily to find new treatments for different kinds of diseases including bacterial infections and especially gastrointestinal disorders. Among these compounds, optically active dihydropyrimidine (DHPM) derivatives have shown a broad spectrum of biological and pharmacological activities (1-5). The medicinal importance of DHPMs such as: antibacterial, antiviral, antitumor, anti-inflammatory and calcium channel blockers activities have been identified (6, 7).

The fast and low-cost synthetic methodologies of novel pharmacologically active compounds are the bottleneck of each drug development. Among these methods, the Biginelli reaction is one of the most applicable multicomponent reactions for the preparation of multi-functionalized 3,4-dihydropyrimidin-2(1*H*)-ones and similar compounds (8-13). Literature survey revealed that, not only the common open-chain *β*-dicarbonyl compounds were used in novel Biginelli-like scaffold synthesis but also cyclic *β*-diketones (14), *β*-ketolactones (15), cyclic *β*-diesters (16) or *β*-diamides (16, 17), benzocyclic ketones and *α*-keto acids were utilized (18).

Urease which is distributed in a variety of organisms is a key enzyme in final step of nitrogen metabolism that catalyzes hydrolysis of urea to ammonia (19). *Helicobacter pylori *plays an important role in gastro duodenal diseases such as gastritis, gastric and duodenal ulcers, and even gastric cancer (20, 21) as well as some other various extraintestinal pathologies. Up to now, many compounds have been proposed as urease inhibitors (22), but some of them are prevented from using *in-vivo* because of their toxicity, poor pharmacokinetic and side effects. On the other hand, natural based compounds are appropriate substitutions for existing inhibitors. In this regard, some isoﬂavones and deoxybenzoins showed urease inhibitory effect (23). Barbiturate derivatives exhibited broad variety of biological effects such as inhibition of MMP-3 (24), MetAP-1 (25), mushroom tyrosinase (26) and they have also antibacterial (27) and sedative (28) properties.

Previously, our efforts to find urease inhibitors resulted in some derivatives with barbiturate based scaffold (29, 30). Besides, other researchers have shown that compounds possessing barbiturate scaffold inhibit urease apart from their biological significance (31, 32). Moreover, *in vivo* studies have confirmed the possible efficacy of barbituric acid on urea splitting activity of gastrointestinal contents of chicks (33). In the present study, thirteen compounds were synthesized and their effect against urease have evaluated.

**Scheme 1:**
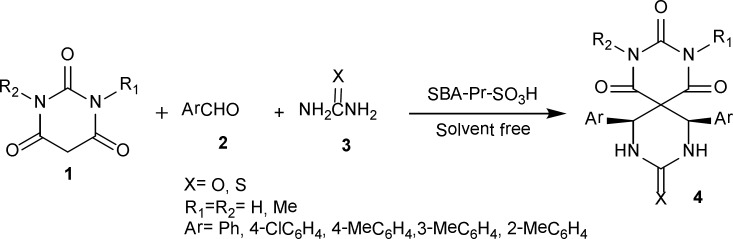
Synthesis of spiropyrimidinethiones/spiropyrimidinones-barbituric acid derivatives 4a-m.

As part of our ongoing program for developing environmentally friendly synthetic methods (30, 34-36), sulfonic acid functionalized ordered mesoporous silica was evaluated as nano acid catalyst (37-39) which mediates Biginelli reaction (Scheme 1). 

Herein we want to report a simple, rapid, one-pot SBA-Pr-SO_3_H mediated synthesis of thirteen spiropyrimidinethiones/spiropyrimidinones-barbituric acids derivatives and their urease inhibitory activities.

## Experimental

***Material and methods***

GC-Mass analysis was measured using the GC-Mass model: 5973 network mass selective detector, GC 6890 Agilent. IR spectra were recorded from KBr disk on the FT-IR Bruker Tensor 27. Melting points were measured using the capillary tube method with an electro thermal 9200 apparatus. The ^1^H-NMR (250 MHz) spectra were recorded on a Bruker DPX, 250 MHz. N_2_ adsorption and desorption isotherms were measured at -196 °C with a Japan Belsorb II system after the samples were vacuum dried at 150 °C overnight. 

***Preparation of catalyst***

The preparation, fictionalization and reusability of the nanoporous SBA-15 were studied based on our previous report (36). The modified SBA-15-Pr-SO_3_H was used as nanoporous solid acid catalyst in the following reaction. 

***General procedure for the preparation of spiroheterobicyclic rings***

The SBA-Pr-SO_3_H (0.02 g) was activated in vacuum at 100°C, and then was cooled to room temperature. In the next step, barbituric acid (0.65 g, 5 mmol), benzaldehyde (1.65 g, 15 mmol) and urea (0.3 g, 5 mmol) were added to the catalyst in a reaction vessel (Scheme 1). The reaction mixture was heated in oil bath at 150 °C for 20 min. After completion of the reaction (indicated by TLC), the catalyst was filtered and the filtrate was cooled to afford the pure solid product. The solid was recrystallized from ethanol, acetonitrile or methanol to afford pure spiro-(2-oxo-4, 6-diphenylhexahydro-pyrimidine-5,5′-barbituric acid). The spectroscopic and analytical data for selected compounds are presented in the following part. The catalyst was washed subsequently with acetonitrile, diluted acid solution, distilled water and acetone, and after drying under vacuum, it can be used for several times without significant loss of activity.

Similarly, the corresponding spiroheterocycles 4b-m were prepared in high yields by the treatment of other substituted aldehydes, barbituric acids or substituted barbituric acids, and urea or thiourea. The results were summarized in the Table 2.

*Spiro-[2-thio-4, 6-di-(3-methylphenyl) hexahydropyrimidine-5, 5′-barbituric acid] *4g

M.p. 236–240°C. IR (KBr) cm^-1^: 3367, 3251 (NH), 2969, 2792, 1715 (CO) 1556 and 1609 (NH Bar bending). ^1^H NMR (250 MHz, CDCl_3_): δ_H_, 2.25 (s, 3H, CH_3_), 2.30 (s, 3H, CH_3_), 5.5 (s, 2H, 2CH), 6.95–7.19 (m, 8H, Ar), 7.85 (s, 2H, NH), 11.02 and 11.36 (2s, 2H, NH) ppm. MS (EI): m/z: 408 (M^+^), 215 (100), 368, 353, 338, 327, 313, 293, 285, 276, 264, 255, 247, 230, 186, 172, 162.

*Spiro-[2-oxo-4, 6-di-(2-methylphenyl) hexahydropyrimidine-5, 5′-barbituric acid] *4h

M.p. 214–216°C. IR (KBr) cm^-1^: 3330, 3193 (NH), 2846, 3061 and 1715 (CO). ^1^H NMR (250 MHz, CDCl_3_): δ_H_, 2.25 (s, 3H, CH_3_), 2.30 (s, 3H, CH_3_), 5.1 (s, 2H, 2CH), 7.1–7.50 (m, 8H, Ar), 8.40 (s, 2H, NH), 11.17 and 11.40 (2s, 2H, NH) ppm. MS (EI): m/z: 392 (M^+^), 215 (100), 368, 361, 353, 339, 327, 313, 299, 285, 276, 265, 257, 239, 230, 172, 142. 

*Spiro-[2-thio-4, 6-di-(2-methylphenyl) hexahydropyrimidine-5, 5′-barbituric acid] *4i

M.p. 224–226°C. IR (KBr) cm^-1^: 3400, 3193 (NH), 2962, 2860 and 1715 (CO). ^1^H NMR (250 MHz, CDCl_3_): δ_H_, 2.25 (s, 3H, CH_3_), 2.30 (s, 3H, CH_3_), 5.45 (s, 2H, 2CH), 7.16–7.57 (m, 8H, Ar), 8.39 (s, 2H, NH), 11.16 and 11.40 (2s, 2H, NH) ppm. MS (EI): m/z: 408 (M^+^), 328 (100), 272, 300, 285, 215, 386, 368, 365, 341.

*Docking approach*

Autodock 4.2 (40), which uses a stochastic search based algorithm, was used for the docking study. AutoDockTools 1.5.4 (ADT) (41) was used to prepare receptor and compounds structures for docking studies by following steps: merge nonpolar hydrogens, adding gasteiger and kollman charges for each compound and enzyme respectively as well as set up rotatable bonds. To evaluate the interaction of each compound in enzyme active site, grid maps and an electrostatic map of each point were calculated. 

Grid calculation was performed by Autogrid 4.2 (40). The parameters were defined 0.375 nm for grid spacing, and each grid map consisted of 40 × 40 × 40 Å points around the active site. Average coordinates of the two Ni^2+^ ions in the α chain of H. pylori urease was set as the center of the grid. A Lamarckian genetic algorithm (LGA) was used which consisted of 250 runs. The initial population considers 150 structures, and the maximum number of energy evaluations sets as 2.5 × 10^7^. The other parameters were set as default values. The final structures were clustered and ranked according to the lowest docking energy.

*Computational resources*

The computational studies were carried out on a computer cluster comprising four sets of HP Prolient ML370-G5 tower servers equipped with two quad-core Intel Xeon E5355 processors (2.66 GHz) and 4 GB of RAM, running a Linux platform (SUSE 10.2).

*Reliability of the docking protocol*

The reliability of the applied docking protocol was determined by re-docking crystallized compound into the active site of the H. pylori urease. If the software could predict binding modes of ligand with receptor, it can used for further studies. To test this, a ligand is taken out of the X-ray structure of its protein–ligand complex and re-docked into enzyme. After comparison of predicted position of ligand in urease active site, the resulted RMSD was 1.42 Å. This protocol was then similarly applied for all synthesized compounds (29).

*Urease inhibition assay*

The biological evaluations were performed by the indophenol method. This is based on the release of ammonia (NH_3_), which reacts with hypochlorite (OCl^−^) to form monochloramine (42). Then the resulted product reacts with phenol to form blue-colored indophenols whose absorbance is measured at 625 nm. 

Briefly, 10 μL of stock enzyme solution (2 mg/mL) was incubated with 140 μL of urea and 5 μL of inhibitor (test compounds) at final concentrations of 1-1.7 mM in phosphate buffer solution (pH 7.6, 100 mM) for 15 min at 37°C. The released ammonia was estimated using equal volume of solution A (containing 5.0 g phenol and 25 mg of sodium nitro prusside) and 500 μL of solution B [containing 2.5 g sodium hydroxide and 4.2 mL of sodium hypochlorite (5% chlorine) in 500 mL of distilled water] at 37°C for 30 min, and the absorbance was measured at 625 nm against the control sample.

## Results and Discussion

This solvent-free one-pot method involves the reaction of barbituric acid 1, benzaldehyde 2 and urea 3 in the presence of nanoporous acid catalyst of SBA-Pr-SO_3_H to afford novel heterobicyclic compounds 4a in good yields (Scheme 1).

Different solvents in the presence of SBA-Pr-SO_3_H as an efficient and recyclable catalyst were screened in the model of reaction. As can be seen in Table 1, in solvent free condition, the desired product, Spiro-(2-oxo-4,6-diphenylhexahydropyrimidine-5,5′-barbituric acid) 4a, was obtained in good yield (Entry 5) and short reaction time (15 min). Our investigations revealed that the best results were obtained in solvent free condition by heating in oil bath at 150°C, therefore these conditions were selected as the optimized one.

**Table 1:** The optimization of reaction conditions in the synthesis of compound 4a.

**No.**	**Solvent**	**Time(min)**	**Yield** ^a^ **(%)**
1	EtOH	30	71
2	EtOH/H_2_O	35	81
3	H_2_O	40	57
4	CH_3_CN	60	80
5	-	15	85

a Isolated yields.

**Scheme 2:**
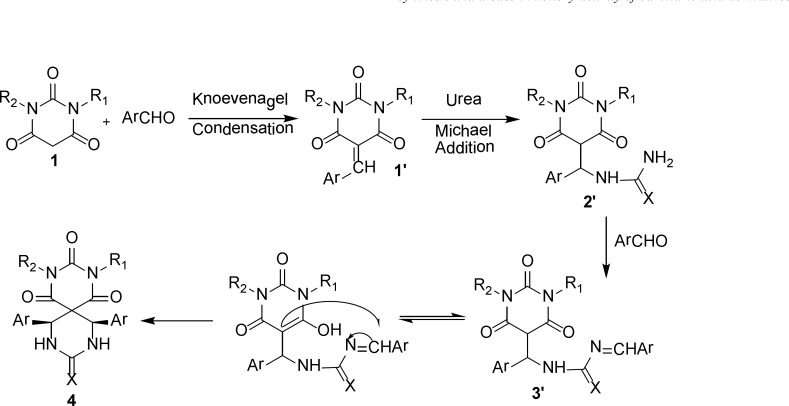
The proposed mechanism for synthesis of 4

The proposed mechanism of product 4 is shown in Scheme 2 as a representative model and the stereochemistry of aryl rings is assigned *cis* (43).

In regard to library construction and evaluation of the substrate scope of this reaction, different barbituric acids, urea or thiourea and aromatic aldehydes were employed under similar circumstances (Table 2). Distinguished utilities of this method are operational simplicity, good yields, and an easy workup protocol without using any chromatographic methods.

**Table 2:** Synthesis of spiropyrimidinethiones/spiropyrimidinones-barbituric acids derivatives

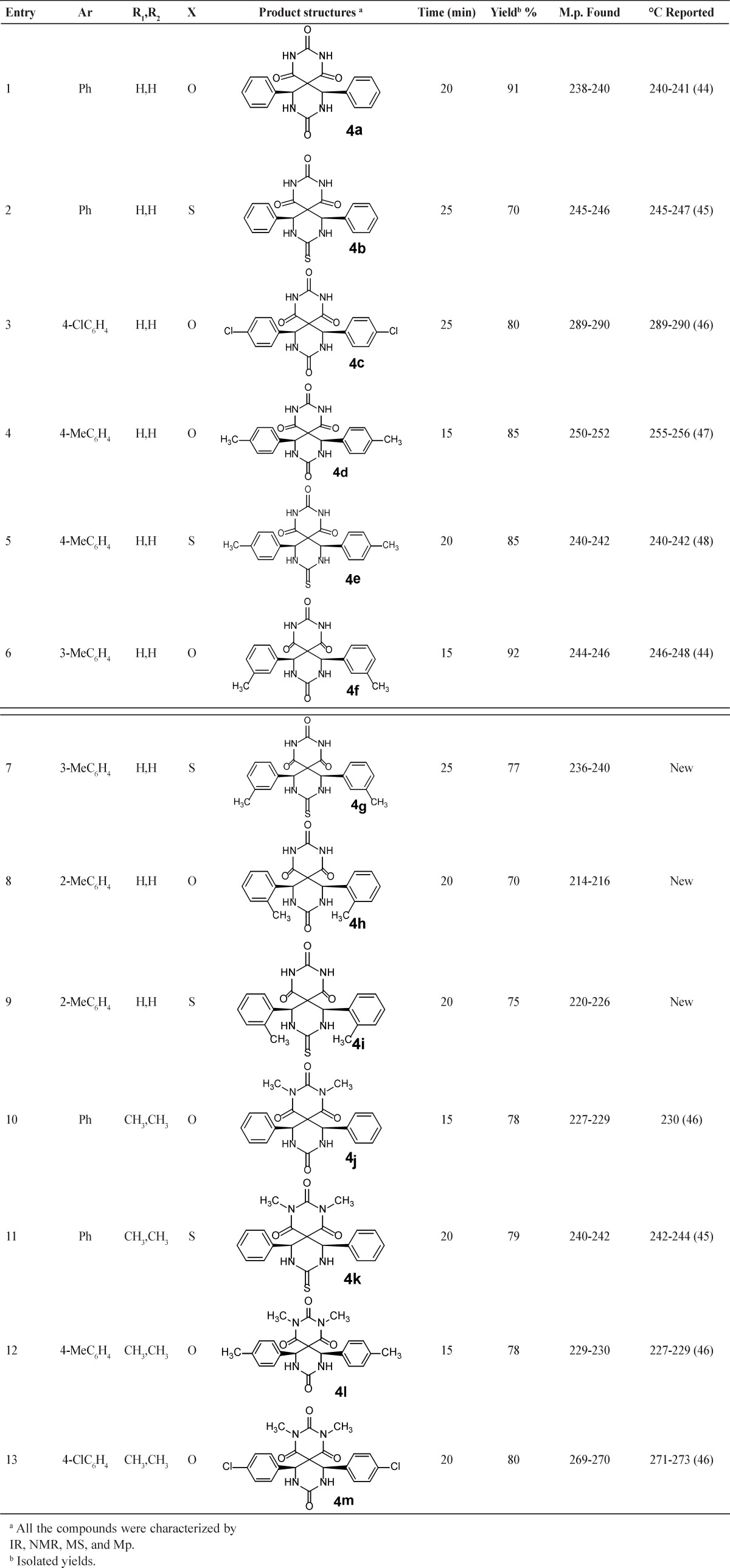

a All the compounds were characterized by IR, NMR, MS, and Mp.

b Isolated yields.

In order to find the best catalyst for the formation of the spiro-fused compounds, we compared the reactions in the presence of various protic liquid or solid acids and Lewis acids such as: 1) In acetic acid/microwave (46), 2) using Iodine/microwave (45), 3) using CoCl_2_/microwave (47), 4) in HCl (49), 5) in acetic acid (44) and NiCl_2_+KI (48) as indicated in Table 3. The results showed that the best yields were obtained in the presence of SBA-Pr-SO_3_H which could acts as nanoreactor.

**Table 3:** The efficiency comparison of various catalysts for the synthesis of 4.

**Entry**	**Catalyst**	**Solvent**	**Condition**	**Time**	**Yield**	**Year**	**Ref.**
1	CH_3_COOH	-	MW	4 min	70-83	2004	(46)
2	Iodine	-	MW	4 min	52-94	2010	(45)
3	CoCl_2_	-	MW	4 min	80-88	2004	(47)
4	-	Acetic acid	Reflux	60 min	57-94	1989	(44)
5	HCl	Ethanol	Heating	24 h	39-90	2003	(49)
6	NiCl_2_+ KI	-	Heating	6 h	80-90	2010	(48)
7	SBA-Pr-SO_3_H	-	Heating	5-30 min	75-92	This work	
8	-	-	Heating	60 min	70	This work	

***Preparation and characterization of catalyst***

**Figure 1:**
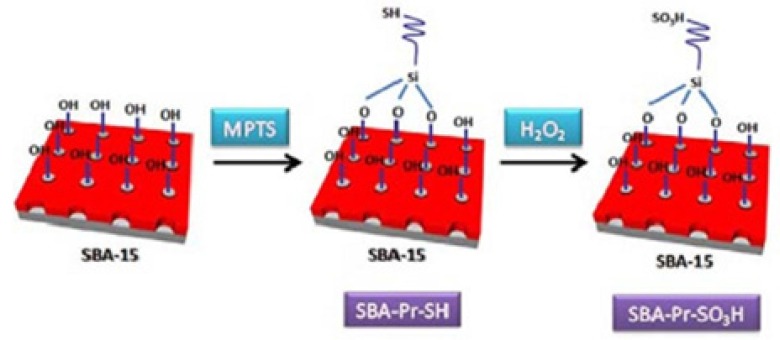
Schematic illustration for the preparation of SBA-Pr-SO_3_H.

The SBA-15 silica was functionalized with (3-mercaptopropyl)trimethoxysilane (MPTS) and then, the thiol groups were oxidized to sulfonic acid by hydrogen peroxide as shown in Figure 1. Analyzing of the catalyst surface was performed by various methods such as TGA, BET, SEM, TEM and CHN methods which was mentioned in our previous work (30).

All prepared products (4) were tested against Jack bean urease and showed the range of inhibition potency, depending on the structural feature (Table 4). Some of synthesized compounds have shown inhibition potency of sub-micromolar through urease. Possibly, the significant enhanced in inhibition of urease potency could be due to stabilization through an additional barbiturate moiety. In this regard, hydrophilic groups interact with the side chains of Lys, and Asp residues surrounding Ni^2+^ ions active site, providing their role in enhancing the potency (50).

**Table 4:** Urease inhbitory activity of spiro-pyrimidinethiones/spiro-pyrimidinones-barbituric acids derivatives

**Product **	**Water solubility**	**Concentration** **(mM)**	**Inhibition (%)**
**4a**	+	1.7	48 ± 0.026
**4b**	+	1.0	2.8 ± 0.046
**4c**	-	1.0	-
**4d**	-	1.7	51 ± 0.052
**4e**	-	1.0	-
**4f**	-	1.0	-
**4g**	-	1.0	-
**4h**	+	1.7	59 ±0.019
**4i**	+	1.0	11 ± 0.042
**4j**	+	1.7	- 42 ± 0.061
**4k**	-	1.0	-13 ± 0.039
**4l**	-	1.0	-
**4m**	-	1.0	-
**Std**	+	0.38	90± 0.23

In most of the derivatives, the oxygen contain derivatives were found to be more potent than the sulphur derivatives which can be explained by their ability to bridge two nickel ions (51). This effect is more apparent in 4a compare to 4b with 48% and 2.8% of inhibition respectively. Moreover, obtained percent of inhibition of compounds 4h and 4i, 59% and 11% approves this effect. Interestingly, in tested compounds 4j and 4k activate enzyme by different potencies, which means that these two compound facilitated urease hydrolysis. Indeed, inserting 2 methyl groups on N atoms of barbiturate ring in 4j, result in totally different effect compared to 4a which may cause to construct more hydrophobic interactions in hydrophobic urease binding pocket (52), however in S substituted compound 4k decrease in activity may due to steric hindrance for S to complex with Ni ions. Indeed, hydrophobic interactions of methyl groups in 4k make longer distance between S and Ni to interact. 

For better understanding of nature and position of substitute, other derivatives were synthesized. According to the obtained results, introduction of electron donating group of methyl in *ortho* position of phenyl ring resulted in increase in activity to 59 % (compare compounds 4h and 4a). Herein, this effect could be explained by better compound stabilization in binding pocket by hydrophobic interactions (53). In compound 4d with *para* methyl substitutes, lowest steric hindrance resulted in 51% inhibition. For investigation of halogen effects in *para* position, corresponded derivatives have been synthesized, but in experienced concentration, none of them showed significant inhibition through urease.

By contrast, irreversible inhibitors showed the time-dependent manner (54). In this series, compounds 4h and 4k accounted as irreversible one that their inhibition varied in 0.5 h and 3 h. Therefore, obtained results motivate our interest in further structural modifications of 4b as a lead compound which provides new template structures for urease in subsequent researches. From all, decline in inhibitor ﬂexibility considerably owing to the rigid aromatic character result in better inhibition which are included in this series and account them as inhibitors for urease.

**Figure 2:**
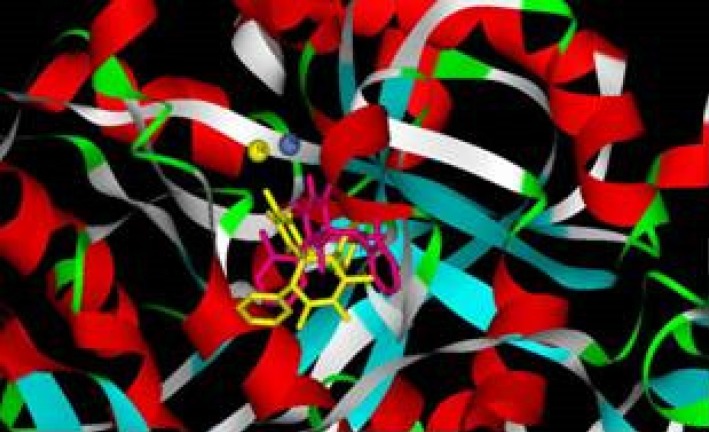
Docking structures of inhibitor (4h, yellow) and activator (4j, pink) of spiro compounds in urease enzyme active site

According to docking studies, in most of the examined compounds, one of the carbonyl groups interacts tightly with both nickel atoms, and the other is involved in the hydrogen bonds with active site residues (Figure 2).

## Conclusions

In conclusion, an efficient, one-pot strategy for the synthesis of spiropyrimidinones-barbituric acids derivatives with urease inhibitory/stimulatory activity using SBA-Pr-SO_3_H as a nano catalyst was reported for the first time. Urease inhibitory activity of spiro compounds was tested against Jack bean urease using Berthelot alkaline phenol–hypochlorite method. For the first time, it was found that five of 13 compounds were inhibitor and two of them were activator.
